# Supplementary material for: Multi-resolution characterization of molecular taxonomies in bulk and single-cell transcriptomics data
Source: Nucleic Acids Res. 2021 Jul 6;49(17):e98. doi: 10.1093/nar/gkab552 (PMC8464061; doi:10.1093/nar/gkab552)
Supplement: gkab552_Supplemental_Files [file gkab552_supplemental_files.zip › supplementary_materials.pdf]

Supplementary Materials

Supplementary Methods

Simulated data generation

K2Taxonomer's performance was assessed in terms of its capability to recapitulate induced hierarchical structure in simulated data, and it was further compared to Ward's agglomerative method as a term of reference. Hierarchically structured data was generated by assigning a mutually exclusive set of *C* labels to *N* observations. To define hierarchical relationships between labels, the set of *C* labels was recursively subdivided into intermediate subgroups until the final subgroups contained only a single label. To represent expected hierarchical structures in real data, we allowed for more than two subgroups to be created at each subdivision. As such, neither *K2Taxonomer* nor agglomerative methods are expected to recapitulate this structure exactly.

After simulating the hierarchical relationships between labels, data was generated as follows. First, a set of 10,000 features was generated, each from a normal distribution, with mean, 0, and standard deviation, BN, denoting the background noise in the data. Second, for each subgroup of labels, we assigned a random set of features for which to add a signal. For each feature assigned to a specific subgroup of labels, the value of the added signal was sampled from a normal distribution with mean, 0, and standard deviation, 2. To ensure that the value of the added signal was the same across all subgroup-specific observations, a feature was only allowed to be assigned to an individual label once. Considering that in real data we expect only a subset of features in a data set contains information of its subgroup structure, prior to

assigning random sets of features to each subgroup, the full set of features was subsetted by a given percentage, DS, of the total.

Simulated data performance assessment

Following the generation of each simulated data set, *K2Taxonomer* and Ward's method were run. The performance of each method was assessed by the relative similarity of the learned structure to the known hierarchy from which the data was generated using Baker's gamma correlation (1). Baker's gamma correlation is a measure of Spearman correlation between two similarity matrices, where each similarity matrix is calculated from the number of shared partitions between each pair observations in a given hierarchy. Baker's gamma correlation ranges between -1 and 1, with 1 representing the case when two dendrograms induce identical hierarchies. This metric was chosen over *cophenetic distance* because *cophenetic* pairwise similarities are calculated using branch heights and branch height is not applicable to the known hierarchy.

Observation-level analysis of simulated data

For observation-level analysis, each simulated data set included N=300 observations. We evaluated the following data generation parameters

- Number of labels (C): 5, 10, 20, 30
- Background Noise (BN): 0.5, 1, 2, 3
- Percent features with signal (DS): 2.5, 5, 10, 25, 50.

Especially when the value of DS is small, Ward's method is not expected to perform well when run on the full set of data. Therefore, Ward's method was evaluated on simulated data with the following variability-based prefiltering percentage levels, PF, of the data:

- Pre-filtering percentage level (PF): 5, 10, 25, 5, 1.

On the other hand, *K2Taxonomer* was always run on the square root of the total number of features. It should be emphasized that this design does not allow for an unbiased comparison of K2T and Ward, and it favors the latter since in real settings we would not know the optimal PF to be used.

Standard deviation was used for variability-based feature selection, including partition-specific feature selection by *K2Taxonomer*. For each combination of these parameters, we generated 25 simulated data sets and ran *K2Taxonomer* and Ward's method. For each combination of parameters, the statistical significance of the difference between the two methods' distributions of Baker's gamma correlation estimates was tested using Wilcox's Rank Sum tests. The resulting p-values from 400 combinations of parameters were corrected for multiple hypotheses testing using the FDR procedure (2).

As noted, for each of these comparisons, we ran *K2Taxonomer* using the square root of the total number of features as the parameter for partition-specific feature filtering. To assess the validity of using this as the default, we performed additional simulations and compared *K2Taxonomer* performance at different partition-specific feature filtering levels, based on the set of percentages of the total number of features: 1%, 2%, 10%, and 20%. These were performed using the same combinations of parameters as the previous analysis and included 25 repetitions.

Group-level analysis of simulated data

For group-level analysis, each simulated data set included N=1000 observations. For data generation, we tested the following sets of data generation parameters

- Number of labels (C): 10, 25, 20, 30
- Background Noise (BN): 0.5, 1, 2, 3
- Percent features with signal (DS): 2.5, 5, 10, 25, 50.

As with observation-level analysis, we performed analyses on simulated data with the following variability-based prefiltering percentage levels, PF, of the data:

- Pre-filtering percentage level (PF): 5, 10, 25, 5, 1.

Linear model-based F-statistics were used for all variability-based feature selection scoring, including partition-specific feature selection by *K2Taxonomer*.

For running each method, the groups were defined by the labels to which each of the observations were assigned. Unlike *K2Taxonomer*, agglomerative methods are not devised to utilize group-level labels for unsupervised learning tasks. Therefore, Ward's method was applied using the Z-score mean value of each group, generated by the same linear model used for feature selection. Comparisons between these methods were performed in the same manner as observation-level analysis.

METABRIC breast cancer primary tumor bulk gene expression processing

METABRIC breast cancer primary tumor Illumina HT-12 v3 microarray bulk gene expression data was obtained from the *CBioPortal*, https://www.cbioportal.org/datasets (3, 4). The data set includes normalized expression values for 24,360 genes and Pam50 cancer subtype estimations for individual primary breast cancer tumor samples across 1,974 female patients. Additional clinical variables considered for this analysis, included: patient age at diagnosis, survival status, ER-status, PR-status, and HER2-status. The distribution of these variables across patients is summarized in Table S1.

TCGA breast cancer primary tumor bulk gene expression data processing

TCGA breast cancer (BRCA) primary tumor bulk RNAseq data was obtained from Genomic Data Commons (GDC*)*, https://gdc.cancer.gov/access-data/ (5). The data set includes raw gene expression counts for 36,812 genes and Pam50 cancer subtype estimations for individual primary breast cancer tumor samples across 973 female patients. Additional utilized clinical variables, included: patient age at diagnosis, ER-status, PR-status, and HER2-status. The distribution of these variables is summarized in Table S2. Raw counts were normalized by the *trimmed mean of M-values (TMM)* method and log-normalized using *edgeR (v3.28.1)* R package (6) and genes with fewer than 2 reads in more than 90% of samples were removed, resulting in 25,729 genes in the processed data set.

Healthy airway tissue scRNAseq gene expression data processing

Publicly available scRNAseq data of normalized, batch corrected, and log-transformed gene expression estimates from airway tissue of healthy subjects, as well as UMAP coordinates, were

obtained from the *UCSC Cell Browser* portal, published as a supplement to the original manuscript for which these data were used, https://www.genomique.eu/cellbrowser/HCA/?ds=HCA_airway_epithelium (7). This data set includes expression estimates for 18,417 genes and 77,969 individual cells from 35 samples across 10 subjects. Multiple samples taken from individual subjects were collected from distinct locations of the human airway including: nasal biopsies, nasal brushings, tracheal biopsies, intermediate bronchial biopsies, and distal brushings. Also, this data set included cell type estimations for each of the 77,969 cells and comprised 28 estimated cell types in total. The methods of data processing, as well as distributions of subject-level sample identities and estimated cell types, can be found in the original publication (7).

References

1. Baker,F.B. (1974) Stability of two hierarchical grouping techniques case 1: sensitivity to data errors. *Journal of the American Statistical Association*, **69**, 440.

2. Benjamini,Y. and Hochberg,Y. (1995) Controlling the false discovery rate: a practical and powerful approach to multiple testing. *Journal of the Royal Statistical Society: Series B (Methodological)*, **57**, 289–300.

3. Pereira,B., Chin,S.-F., Rueda,O.M., Vollan,H.-K.M., Provenzano,E., Bardwell,H.A., Pugh,M., Jones,L., Russell,R., Sammut,S.-J., *et al.* (2016) The somatic mutation profiles of 2,433 breast cancers refine their genomic and transcriptomic landscapes. *Nat Commun*, **7**, 11479.

4. Cerami,E., Gao,J., Dogrusoz,U., Gross,B.E., Sumer,S.O., Aksoy,B.A., Jacobsen,A., Byrne,C.J., Heuer,M.L., Larsson,E., *et al.* (2012) The cBIO Cancer Genomics Portal: an open platform for exploring multidimensional cancer genomics data. *Cancer Discovery*, **2**, 401–404.

5. The Cancer Genome Atlas Network (2012) Comprehensive molecular portraits of human breast tumours. *Nature*, **490**, 61–70.

6. Robinson,M.D., McCarthy,D.J. and Smyth,G.K. (2010) edgeR: a Bioconductor package for differential expression analysis of digital gene expression data. *Bioinformatics*, **26**, 139–140.

7. Deprez,M., Zaragosi,L.-E., Truchi,M., Becavin,C., Ruiz García,S., Arguel,M.-J., Plaisant,M., Magnone,V., Lebrigand,K., Abelanet,S., *et al.* (2020) A Single-cell Atlas of the Human Healthy Airways. *Am J Respir Crit Care Med*, 10.1164/rccm.201911-2199OC.

Supplementary Figures

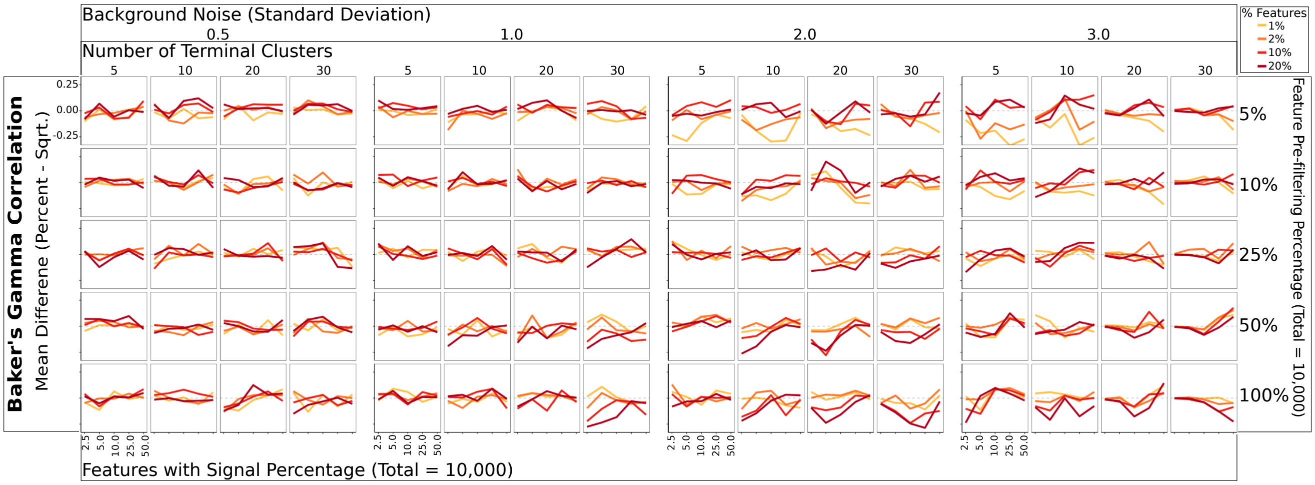

Figure S1: Simulation-based performance assessment of running *K2Taxonomer* using different partition-specific feature subset sizes of the full data set

Mean Baker's gamma correlation estimates measuring the similarity of *K2Taxonomer* inferred hierarchies to the true hierarchy from which the simulated data were generated. Each line shows the difference between a set percentage of the total number of features and the square root of the total number of features. Each combination of parameters was simulated 25 times.

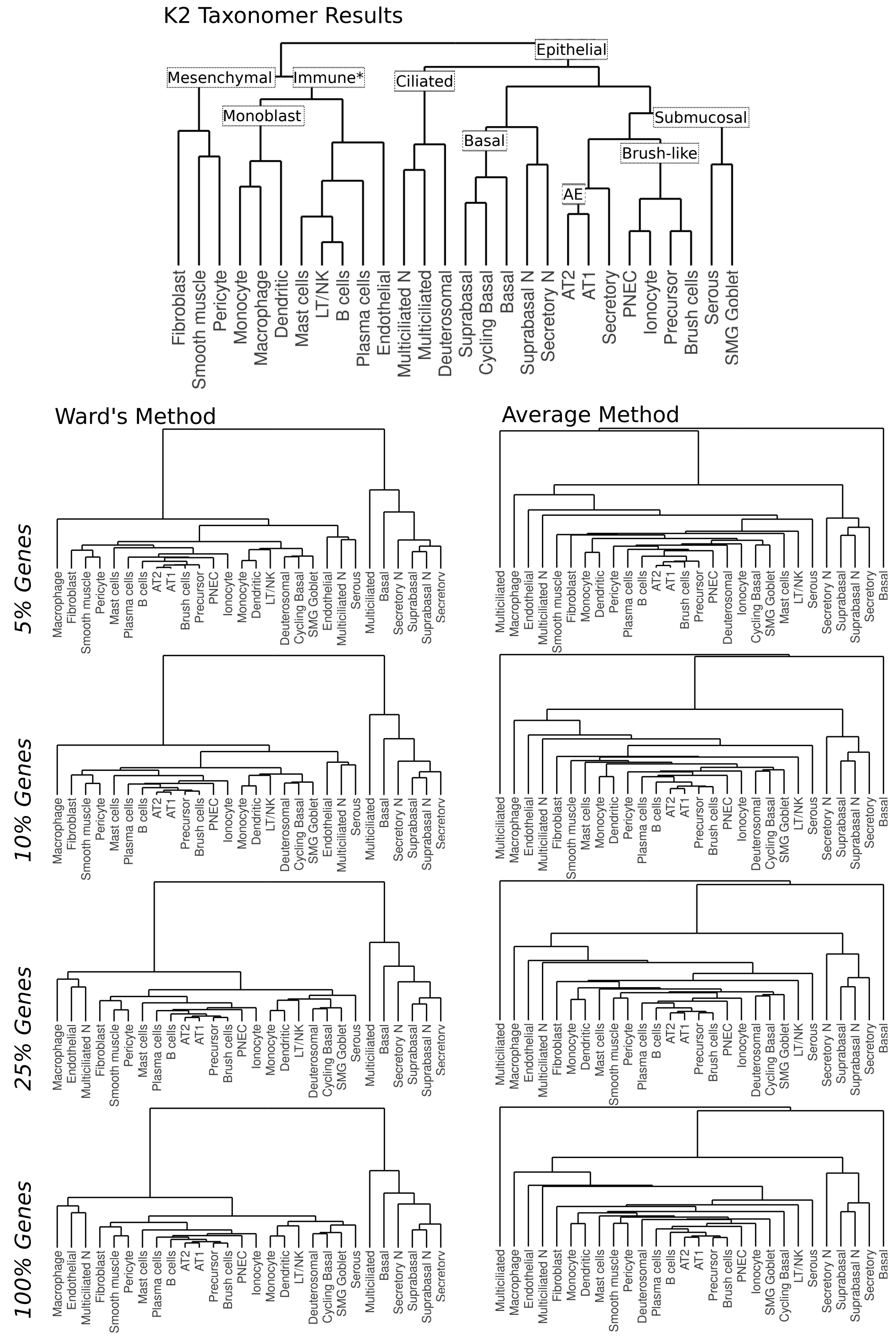

Figure S2: Additional subgrouping results of healthy airway cell types from scRNAseq data

Ward’s (left) and average (right) agglomerative clustering results for analyses performed on different subsets of the total number of genes.

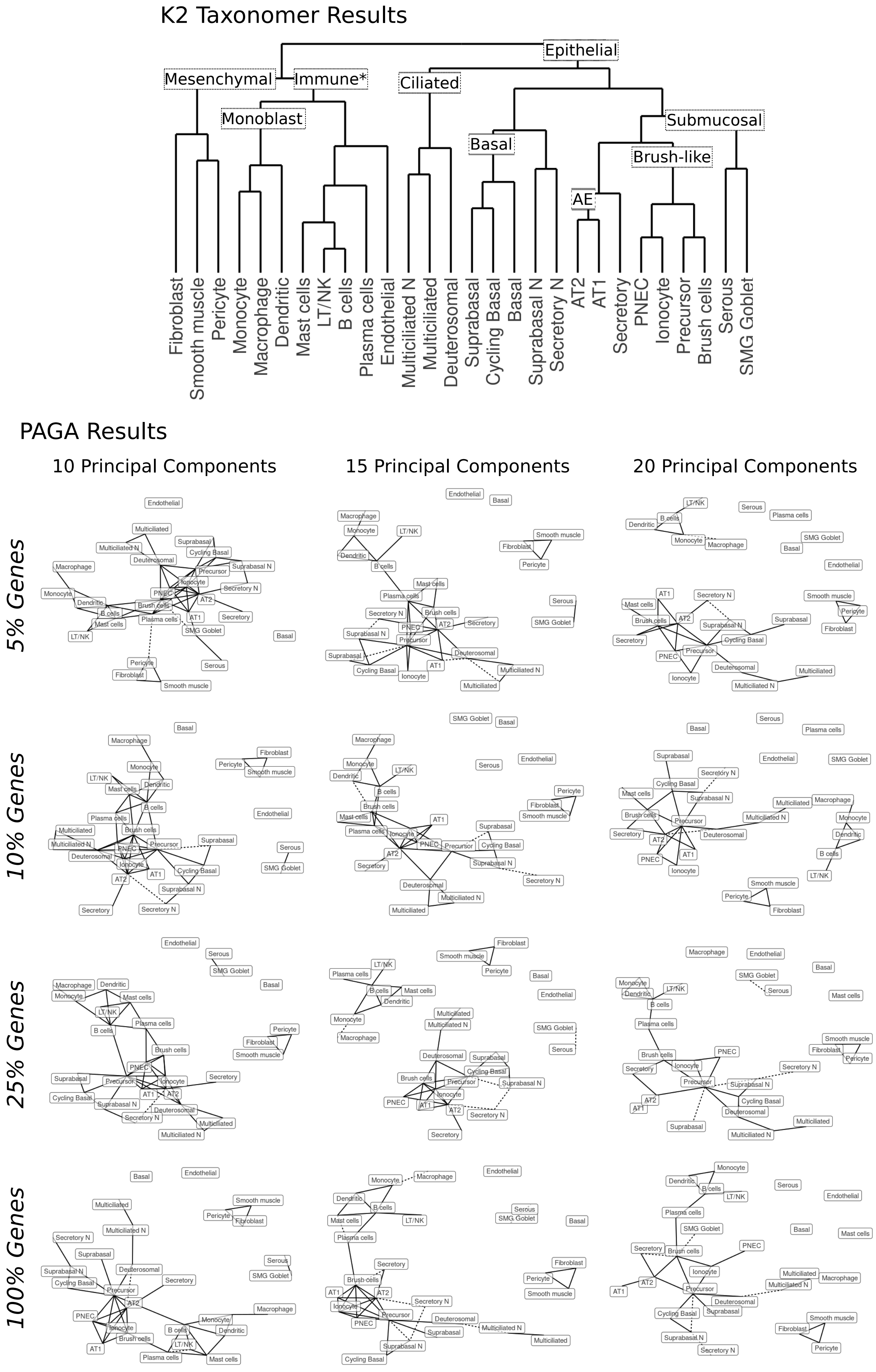
K2 Taxonomer Results
Epithelial
Mesenchymal
Immune*
Ciliated
Monoblast
Submucosal
Basal
Brush-like
AE
Fibroblast
Smooth muscle
Pericyte
Monocyte
Macrophage
Dendritic
Mast cells
LT/NK
B cells
Plasma cells
Endothelial
Multiciliated N
Multiciliated
Deuterosomal
Suprabasal
Cycling Basal
Basal
Suprabasal N
Secretory N
AT2
AT1
Secretory
PNEC
Ionocyte
Precursor
Brush cells
Serous
SMG Goblet
PAGA Results
10 Principal Components
15 Principal Components
20 Principal Components
5% Genes
10% Genes
25% Genes
100% Genes

Figure S3: Additional *PAGA* graph-based trajectory results of healthy airway cell types from scRNAseq data

PAGA graph-based trajectory results for analyses performed on different combinations of numbers of principal components and subsets of the total number of genes. The annotated model returned by *K2Taxonomer* is shown above the *PAGA* model graphs.

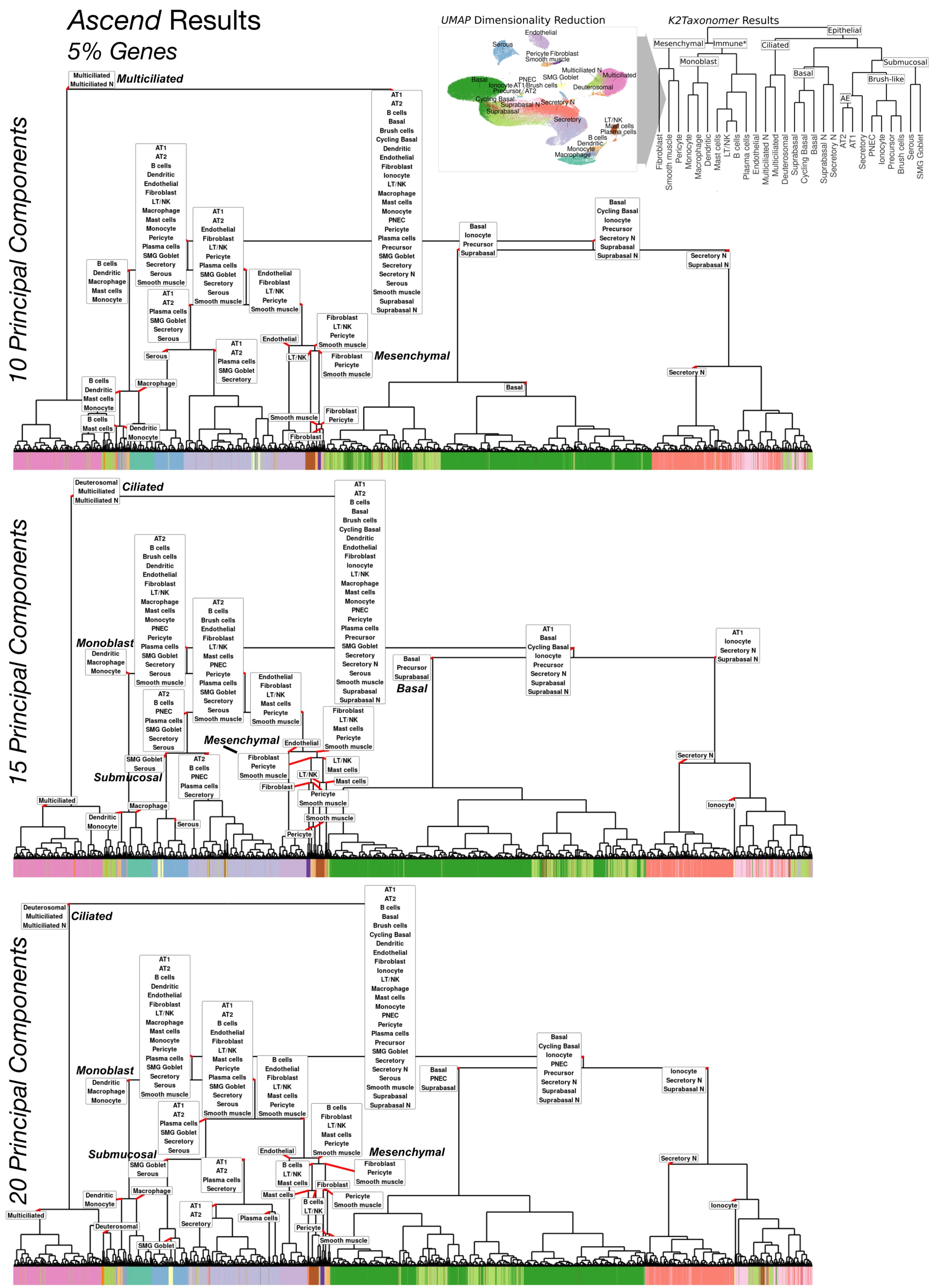

Ascend Results
5% Genes
UMAP Dimensionality Reduction
K2Taxonomer Results
10 Principal Components
15 Principal Components
20 Principal Components
Multiciliated
Ciliated
Mesenchymal
Basal
Monoblast
Submucosal
Epithelial
Immune*
Brush-like
AE

Ascend Results
10% Genes

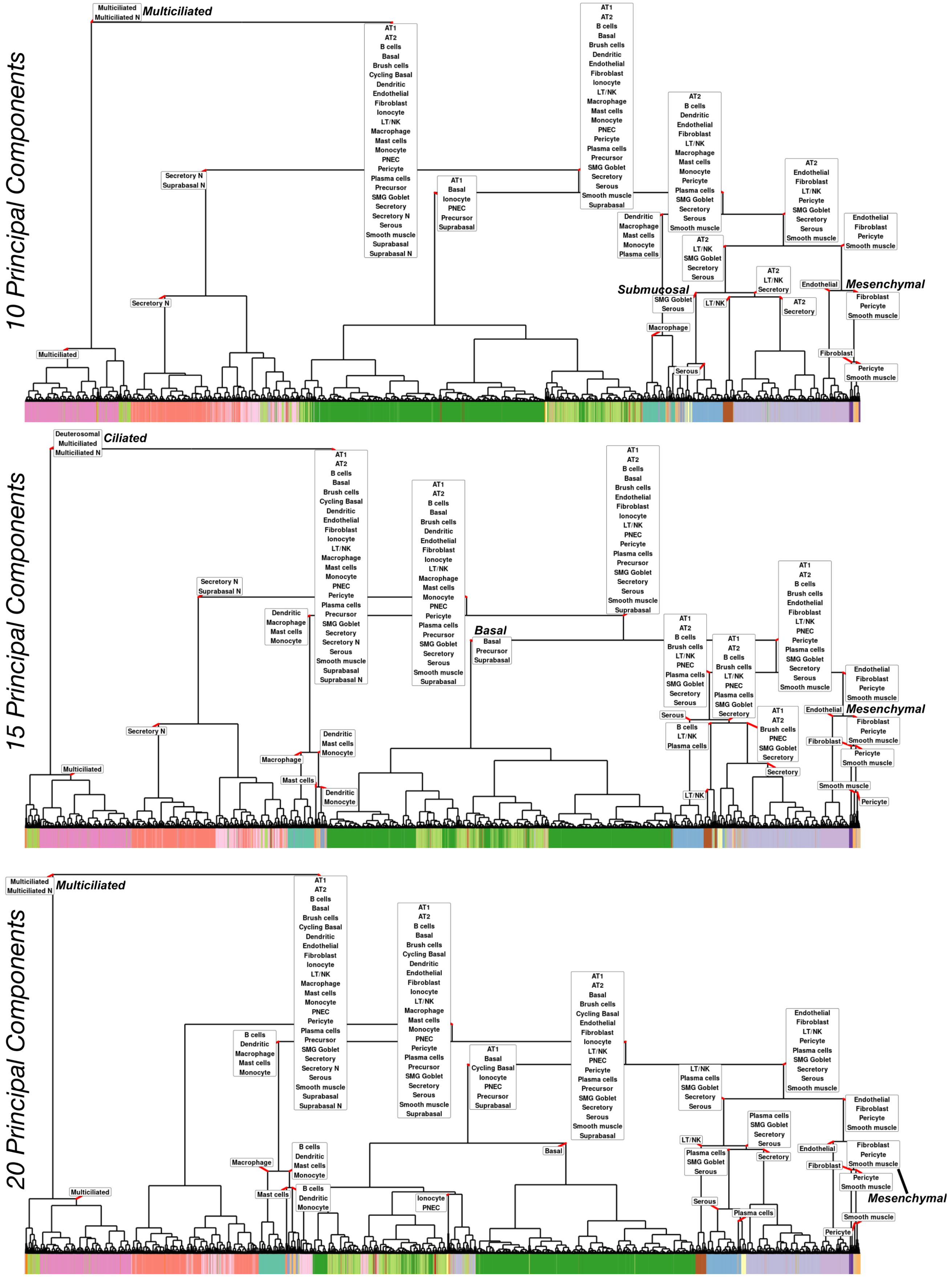

Ascend Results
25% Genes

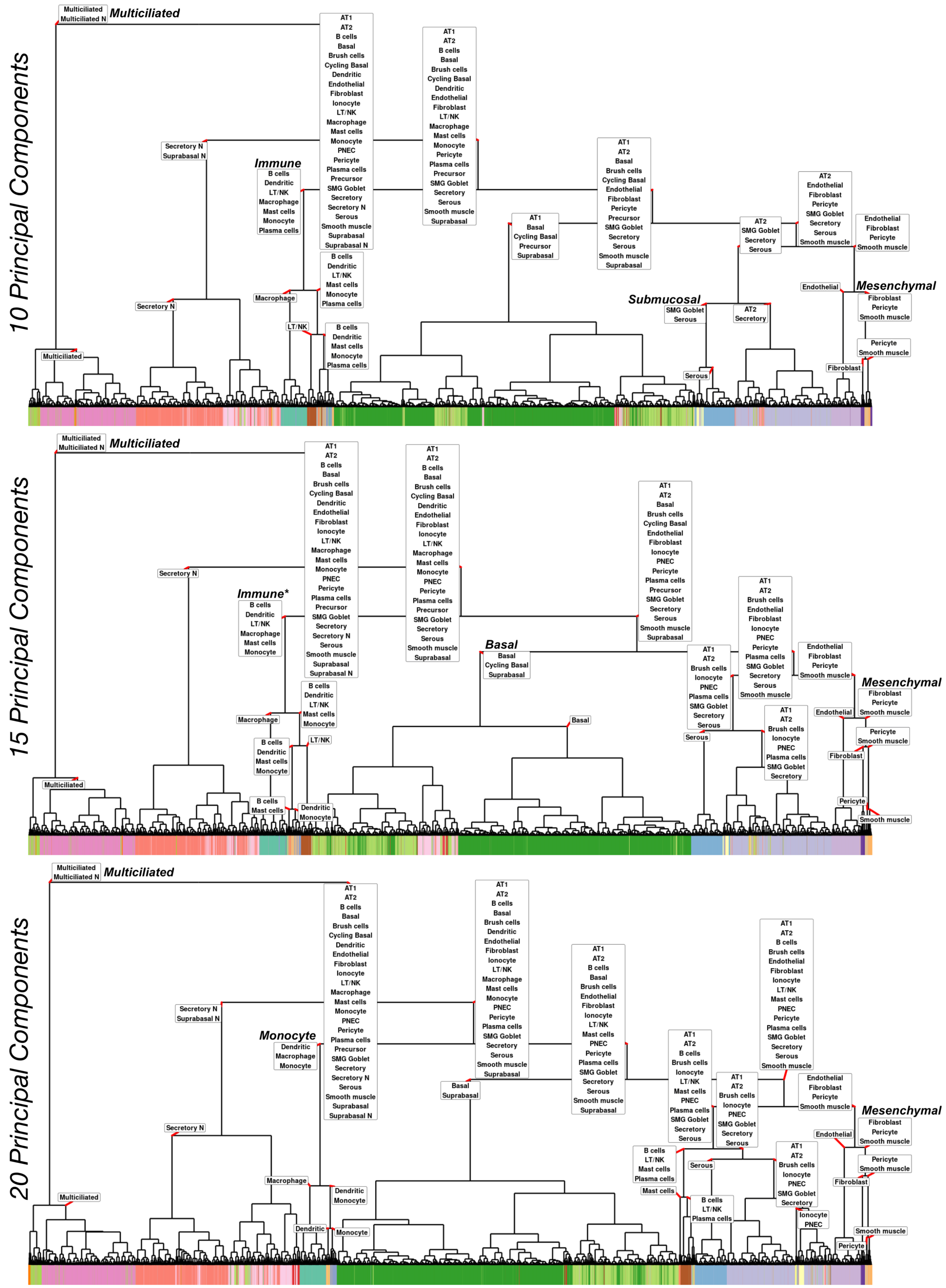

Figure S4: *Ascend* scRNA-seq hierarchical subgrouping results of healthy airway cell types from scRNAseq data

Ascend scRNA-seq hierarchical subgrouping results for analyses performed based on different combinations of numbers of principal components and subsets of the total number of genes. The annotated model returned by *K2Taxonomer* and the plot of cell clusters labelled "*UMAP* dimensionality reduction" are shown above the *ascend* model dendrogram. Colors below the *Ascend* dendrogram indicate the cluster labels and are consistent with that of the *UMAP* dimensionality reduction plot. Intermediate labels in the *ascend* model were assigned if they satisfied two criteria: first, if at least 75% of the profiles of individual cell types were assigned to the subgroup and, second, if the summation of the cell profiles from individual cell types that met the first criteria made up at least 75% of the total cell profiles in the subgroup. The "*" in the "Immune" subgroup label for the 25% genes + 15 principal components *ascend* model indicates the incompleteness of this subgroup caused by the absence of plasma cells.

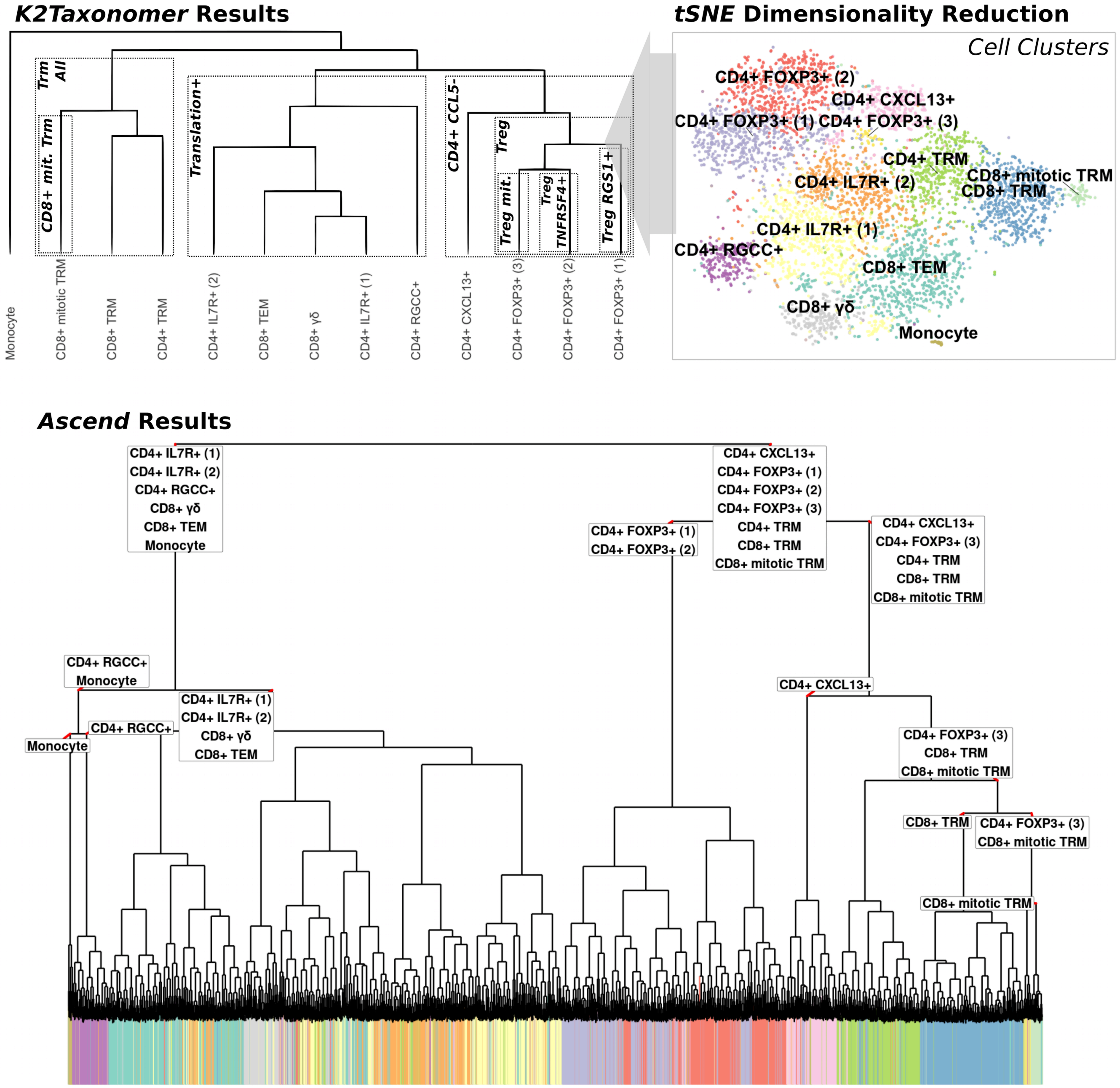

Figure S5: *Ascend* scRNA-seq hierarchical subgrouping results of breast cancer immune cell data

Ascend scRNA-seq hierarchical modeling was performed on the same dimensionality reduction matrix used for *Seurat* "flat cluster" discovery. The annotated model returned by *K2Taxonomer* and plot of cell cluster labelled tSNE dimensionality reduction plot are shown above the *ascend* model dendrogram. Colors below the Ascend dendrogram indicate the cluster labels and are consistent with that of the *tSNE* dimensionality reduction plot. Intermediate labels in the *ascend* model were assigned if they satisfied two criteria: first, if at least 75% of the profiles of individual cell clusters were assigned to the subgroup and, second, if the summation of the cell profiles from individual cell clusters that met the first criteria made up at least 75% of the total cell profiles in the subgroup.
